# Shades of digital deception: Self-presentation among men seeking men on locative dating apps

**DOI:** 10.1177/13548565221102714

**Published:** 2022-05-16

**Authors:** Eric Filice, Corey W Johnson, Diana C Parry, Harrison Oakes

**Affiliations:** School of Public Health and Health Systems, 8430 University of Waterloo, Canada; Department of Recreation and Leisure Studies, 8430 University of Waterloo, Canada; Department of Recreation and Leisure Studies, 8430 University of Waterloo, Canada; Department of Psychology, 8430 University of Waterloo, Canada

**Keywords:** mobile media, internet-mediated communication, dating apps, self-presentation, impression management, sexuality

## Abstract

In recent years, location-based real-time dating apps like Grindr and Tinder have assumed an increasingly pivotal role in brokering socio-sexual relations between men seeking men and have proven to be fertile ground for the study of identity negotiation and impression management. However, current research has given insufficient consideration to how various contextual elements of technology use interact with one another to shape self-presentation behaviour. Through analysis of interview data, we found impression construction on these apps reflects tensions between authentic depiction of the self-concept and self-enhancement via deception. Whether and the extent to which one engages in deception depends on how a number of technological affordances, platform-specific community norms and userbase characteristics interact with each other. Self-presentational choices were a result of a combination of deception facilitators, for example, belief in the normalcy of lying, and constraining determinants, for example, the expectation of brokering physical connection. Impression construction determinants also interact in ways where the influence of any one element is dependent on others. This was most plainly evidenced in the interactions between stigma management concerns, the affordances of audience visibility/control and locatability and common ground reinforcing social hierarchy.

In recent decades, networked technologies have assumed an increasingly pivotal role in brokering interpersonal connections of a romantic, sexual or otherwise intimate character. In 2019, 30% of US adults reported having previously used an online dating Web site or mobile dating app, up from 11% in 2013 (Anderson et al., 2020). Non-heterosexuals are especially keen adopters of such technologies, with approximately four-in-10 individuals in same-sex relationships (37%) reporting having met their partner online compared to 11% of individuals in different-sex relationships (Brown, 2019). As others have noted, there is social and historical precedent for the vigorous uptake of dating technologies by sexual minorities, particularly gay, bisexual and other men who have sex with men (GBMSM). Since same-sex attraction is both (a) culturally stigmatized and (b) an invisible or only partly visible trait, the internet has long been valued as a means of identifying other GBMSM and communicating one’s sexual or romantic intentions with lesser risk of harassment, violence or arrest, in much the same way as the ‘handkerchief code’ deployed in the pre-digital era (Birnholtz et al., 2014; Grov et al., 2014; Gudelunas, 2012).

Dating technologies themselves have undergone significant transformation in recent years. In the first decade of the new millennium, online dating mostly took place through more traditional ‘web-based dating sites’ (Gudelunas, 2012), like Match, eHarmony and OkCupid. These sites were tethered to personal computers and connected individuals only within a general geographic area, usually manually inputted by the user and not based on any finer degree of proximity (Blackwell et al., 2015). Since then, a class of software for mobile devices sometimes referred to as location-based real-time dating apps (LBRTDAs) (Handel and Schlovski, 2012) have gained considerable traction. According to Miles (2018), LBRTDAs are distinguished by their convergent use of spatial coordinates, mobile signal and satellite position via GPS to situate users with cartographic specificity. Users are then typically displayed to each other en masse in a social grid by order of distance. This is intended to both reduce the time spent searching for potential matches as well as shorten the distance between users, thereby expediting relationship initiation and transition to offline, face-to-face encounter (Miles, 2017), as reflected in the apps’ technological infrastructure and marketing which ‘communicate a narrative of immediacy and efficiency’. (Miles, 2018: 6) Though some LBRTDAs are popular among heterosexuals (e.g., Tinder, Bumble), these apps have a longer history of use among GBMSM. Grindr is widely considered the first ever LBRTDA. Originally released in 2009, it is expressly marketed for use by ‘gay, bi, trans, and queer people’ (Grindr, 2020). A recent survey of over 3000 GBMSM revealed Grindr to be the most frequently used app, with 60.2% of the sample reporting some or frequent use. Other widely-used GBMSM-targeted LBRTDAs include Jack’d, SCRUFF (targeted towards ‘bears’ and hairier men) and Recon (for men interested in fetish and kink) (Badal et al., 2018).

Many of the most common LBRTDAs present users with a grid or list of profile thumbnails that are arranged in order of geographic proximity to the active user. To access a profile, users click on the thumbnail. From the profile, users can write each other text messages or send a preprogrammed message via one of 3 ‘taps’ on Grindr (i.e., ‘Hi there!’ ‘You’re hot,’ ‘Are you looking [for sex]?’), a ‘Woof’ on SCRUFF or ‘cruising’ a profile on Recon. Across Grindr, SCRUFF and Recon, users are afforded many similar markers for profile construction (e.g., display name, geographical distance from other users, online status, demographics, sexual information [e.g., position preference, safe sex practices], relationship status, intent [e.g., chats, friendship, casual sex]), but also certain app-specific options. For example, Grindr users can indicate their gender and pronouns, SCRUFF users can indicate communities (e.g., queer, college, jock) of interest and Recon users can select 5 (of 26) kink interests. Users on Grindr and SCRUFF can also link their profiles to other social media accounts (e.g., Facebook, Instagram and Twitter). Beyond categorical identity markers, all three GSNAs also provide users open-ended fields to describe themselves, which differ in the amount of allotted space.

LBRTDAs’ pervasiveness in contemporary gay male socio-sexual relations (i.e., ‘hybrid’ forms of interpersonal connection that simultaneously occupy the realms of the platonic, erotic and practical; Shield, 2018) has spurred inquiry into their influence on various social-psychological phenomena, including ‘risky’ sexual behaviours (Goedel and Duncan, 2016), relationship development and maintenance (Licoppe et al., 2016; Møller and Petersen, 2018; Race, 2015) and community structure and dynamics (Miles, 2017; Mowlabocus, 2010). Another area that has captured researchers’ interest, and is the focus of the present study, is identity negotiation and presentation of the self. It is well established that self-presentation plays an important role in relationship development, and thus is pertinent to dating technologies. Particularly in the early stages of relationship initiation, individuals rely heavily on impressions others generate to determine if they wish to pursue deeper levels of connection (Derlega et al., 1987; Taylor and Altman, 1987). Research suggests that individuals are cognizant of this fact, and on first dates will alter their behaviour to conform with what they assume are the values held by their potential companion (Rowatt et al., 1998). In the present study, we use narrative interview data to explore how various features and contingencies of LBRTDA use interact to shape GBMSM’s self-presentation behaviour, including facets of the communication technology itself, individual user idiosyncrasies and the encompassing social context. First, we present an overview of the literature informing our theoretical framework.

## Background

### Symbolic interactionism and identity negotiation

Scholarship on technology-mediated construction and display of identity has traditionally been undertaken either from the vantage of symbolic interactionism or postmodernism-poststructuralism, which differ primarily with respect to how they make sense of the ‘performative interval’ that interposes the subject’s sense of self and social action. Whereas interactionists treat this discontinuity as a springboard to investigate how individuals, in pursuit of consolidating a more-or-less stable interiority, attempt to bridge the divide between expressions and the identities they signify, poststructuralists focus on the disjunct between these poles – or individuals’ performative *failures* – to evidence a purely discursive self devoid of such an interiority (Green, 2007). For postmodernists and poststructuralists, who reject interactionist notions of a socially-derived master-self, virtual environments are quintessential ‘liminal spaces’ wherein identity displays are decoupled from their embodiment, relieved of the constraints of corporeal warrant, allowing for greater experimentation and creative play (Lupton, 1995; Stone, 1996; Turkle, 1995). As Robinson (2007) critiques, however, these suppositions are somewhat of an anachronism based on the backgrounds and usage patterns of modern internet users. Whereas the typical user in the 90s was a white, affluent man with a penchant for fantasy role-playing games, today’s users are more heterogeneous in terms of race, socioeconomic status and gender (Duggan and Brenner, 2013), and are oriented to technology more as a way to augment and extend, rather than escape, offline life (Kennedy, 2006; Subrahmanyam and Śmahel, 2010). We hence echo Robinson’s (2007) assertion of the continued relevance of interactionist conceptions of self, and by extension dramaturgical analyses of self-expression, detailed in the following section.

### Self-presentation

Self-presentation refers to the goal-directed activity of controlling information in order to influence the impressions others form of oneself (Schlenker, 2012). In his original formulation, Goffman (1959) argued that individuals almost always pursue interaction in light of an ultimate objective and/or series of underlying motives, consciously or otherwise. This can include spurring an observer to perceive oneself in a certain way, ensuring sufficient interactional harmony to sustain relationships or maximizing the social and material rewards of interaction. To that end, individuals will try to impress upon audiences a particular ‘definition of the situation’ (Goffman, 1959: 4) that would lead them to act voluntarily in ways conducive to the fulfilment of said goals. Because the nature of social reality is such that it can only be inferred to a limited degree of accuracy from communicative gestures, it is not incumbent that these impressions be incidental to or an uncontrived expression of a certain ‘real’ state of affairs for them to achieve their intended effects – the impression fostered need only convince observers of such. Thus, when an individual stages a performance they entreat observers to accept the version of reality offered, including claims as to the true nature of oneself. Of most central concern, then, is whether one’s performance will be credited or discredited. The primary means audiences have of establishing the credibility of a performance is evaluating internal consistency by cross-checking expressions ‘given’ against those ‘given off’. Expressions given are signifying acts over which the performer has conscious control and can manipulate with relative ease, most usually verbal assertions. Conversely, expressions given off are those that evade conscious awareness or are difficult to control by the performer and thus unwittingly ‘leak out’, like nonverbal expressions and appearance. The successful performer must ensure any self- or reality-claims made are not contradicted by their actions or contextual information, lest they be revealed a fraud (Goffman, 1959).

Despite Goffman’s exhaustive cataloguing of the techniques employed in impression management, Leary and Kowalski (1990) found his framework to be lacking any systematic consideration for the antecedent factors that motivate self-presentation and shape its content. To remedy this, they propose a model separating self-presentation into two distinct processes: impression motivation, or the reasons why people choose to engage in impression management and the factors affecting their drive to do so, and impression construction, or the factors that inform the kind of impression one chooses to generate and how they go about it. Alongside established factors such as self-concept, social norms and audience values, Toma and Hancock (2010) advise communication medium be added to Leary and Kowalski’s (1990) two-component model as a determinant of impression construction. However, we still lack a mechanistic explanation as to how impression management and communication medium are linked. Borrowing from DeVito et al. (2018b), we posit this link occurs in the context of a social-technological ‘ecosystem’ that is constituted through the reciprocal interactions between platforms’ technological affordances, behavioural norms, the individual user and the presentation-relevant social context. We describe these components in turn.

### Affordance

Most fundamentally at issue here is how a particular class of technologies (LBRTDAs) influence a specific social process (self-presentation). Implicit to any derivation of this basic question are assumptions of how human and material agencies relate to one another. This includes the degree to which the properties we observe of material artifacts like networked technologies are intrinsic to them or are constituted by collective acts of meaning-making, as well as the level of autonomy humans and non-human artifacts have relative to one another (Faraj and Azad, 2012; Leonardi, 2011). The affordance lens is lauded for recognizing the mutually constitutive relationship between material and social aspects of technology and sufficiently balancing tensions between technological determinism and voluntarism (Faraj and Azad, 2012; Orlikowski, 2007), which accords well with the symbolic interactionist belief in humans’ capacity to act in a conscious, willful and strategic matter even when subject to external forces (Blumer, 1969; Snow, 2001). Faraj and Azad (2012, in Majchrzak et al., 2013) define affordances as ‘the mutuality of actor intentions and technology capabilities that provide the potential for a particular action’. In this sense, affordances are not an attribute possessed by actors nor technologies, but an outcome of the relational dynamics between the two. The benefits to examining technology-related social change through the lens of affordance are numerous. First and foremost, the affordance lens allows for high-level analysis of capabilities that technologies provide users in a way that is not restricted to any specific software/hardware or version. Additionally, because this lens transcends any one particular technological form, iteration or context of use, the findings produced may still be of theoretical importance even after such technologies have undergone dramatic change (Ellison and Vitak, 2015).

### Communal common ground

We have established that one of the key means by which networked technologies influence impression construction is through changes to users’ self-presentational *capabilities* made possible by the perceived affordances of these technologies. Another element, Ellison et al. (2011) suggest, is the shared contextual knowledge and expectations among collectives of users that shape normative lines of action. Clark (1996) refers to the shared stocks of background knowledge, assumptions, values, procedures and lexicons that are relied upon for meaningful interaction and assumed to be known to any member of a given collective as its ‘communal common ground’. Researchers have long maintained that online platforms can be host to groups of users that collectively negotiate specific communal common ground, as evidenced by popular concepts like ‘netiquette’ (Scheuermann and Taylor, 1997), which describe conventions of politeness unique to networked environments. Previous research has illustrated how shared expectations of normative behaviour in online spaces inform techniques of self-presentation. For example, online daters tend to embellish certain personal details in their profiles in part because they assume others are doing the same (Ellison et al., 2011; Fiore and Donath, 2004; Jaspal, 2017). This observation is illustrative of the logic that inspires our inclusion of communal common ground alongside affordance in examining mediated self-presentation – namely, that it is as much an issue of what users are *able* to do as it is what they *expect* and tolerate of each other.

It should be noted that our invocation of communal common ground is not meant to substitute for the concept of community norms, as this would be to assume, potentially incorrectly, that the aggregate of users on gay male LBRTDAs necessarily see themselves as constituting a community. Miles (2017) demonstrates that while gay male LBRTDA users are relatively unified in their understanding of community as a group of like-minded people who have something in common, they were ambivalent as to whether this was something that actually manifested in practice online, some going as far as to frame Grindr, contrariwise, as an ‘anonymous, self-serving public’. (Miles, 2017: 1601) Moreover, to treat community – and the degree of social cohesiveness it implies – as self-evident on gay LBRTDAs belies the extent of stratification and marginalization on these platforms. An abundant literature attest to the pervasiveness of sanctioning and exclusion on the basis of race (Daroya, 2018; Robinson, 2015), ability (Shield, 2018), gender (Lloyd and Finn, 2017) and appearance (Conner, 2019; Filice et al., 2019).

### Individual- and context-related factors

Another factor that informs self-presentation decisions on digital platforms is the user themselves and the way they are situated within the broader social context. These elements can be viewed as the psychological and social preconditions that dispose users to present themselves in certain ways online. To wit, researchers have identified a number of individual-level characteristics that bear on self-presentation behaviour in mediated environments, including interpersonal skill (e.g., degree of self-awareness in public, ability to notice and interpret the behaviours, thoughts and feelings of others), motivation (e.g., level of concern for public approval), goals of technology use and technological competency (i.e., the ability to grasp the available tools of a platform and use them to accomplish one’s goals) (Litt, 2012). Demonstrating this point in the context of gay male LBRTDAs, Bonner-Thompson (2017) found Grindr users’ choice in presentation style was guided in large part by their motivations – ‘hypersexualized’ displays (i.e., emphasis on the body with full or partial nudity), for instance, were commonly deployed by those aiming to signify sexual availability and stimulate tactile desire in others. Similarly, Miles (2019) identified a series of ‘practice-based identities’ which are differentially mobilized depending on users’ level of experience and incentive to broker physical encounter.

Individual differences in self-presentation behaviour do not simply reflect variations in agents’ isolated dispositions and predilections, however. As many rightly note, technology users are embedded in broader social, cultural and historical conditions which shape their general lived experience and, in turn, contingencies of use (Baumer and Brubaker, 2017; DeVito et al., 2017). As Duguay (2016) suggests, one such socio-historical configuration which collectively defines the condition of contemporary sexual minority persons, and which therefore merits focused inquiry into their experiences of mediated self-presentation, is the possession and need to negotiate disclosure of a stigma. Goffman (1963) argues that identities have a moral dimension insofar as they elicit normative judgments from others. Stigma thus refers to attributes of an individual which, as a consequence of routinized forms of reason and meaning (elsewhere defined as dominant ideology (Marx and Engels, 1998 [1845]) or power-knowledge complexes (Foucault, 1978)), are judged to be abnormal, inferior or intolerable, and which thereby discredit the identity claims made by their possessors in social situations. Scott (2015: 156) notes, ‘The normal and the stigmatized are not essential but relational identities, each defined by contrast to the other. It is the disjuncture between normative expectations and perceived difference that creates the perception of stigma in others’. As such, impression management strategies for those with discreditable stigmas such as deviation from cisgender heterosexuality often prioritize concealing the attribute and preventing its inopportune revelation to unsympathetic audiences (Goffman, 1963; Scott, 2015).

### Framing the present study

It hence stands to reason that mediated self-presentation behaviour will change alongside technologies, users and contexts. In light of this, self-presentation researchers’ focus has shifted in recent years from more traditional web-based dating sites (e.g., Ellison et al., 2006; Gibbs et al., 2006; Guadagno et al., 2012; Manning, 2013; Toma and Hancock, 2010; Toma et al., 2008) to LBRTDAs (e.g., Duguay, 2017; Ranzini and Lutz, 2017; Ward, 2017) with the gradual supplanting of the former technology by the latter. An additional literature, acknowledging the potential influence of sexual identity on impression construction, specifically examine GBMSM’s self-presentation on online dating technologies (Birnholtz et al., 2014; Blackwell et al., 2015; Bonner-Thompson, 2017; Cassidy, 2013, 2016; Chan, 2016; Jaspal, 2017; Light et al., 2008; Miller, 2015, 2018). The current study seeks to extend this line of work while innovating theoretically and methodologically. Specifically, one persistent issue in the extant literature is the focus on the *content* and *strategies* of identity display on gay male LBRTDAs to the neglect of the determinants of their construction – in other words, we have a good idea of how GBMSM present themselves on LBRTDAs but only a limited understanding as to why. This is partly a consequence of the limitations of the current procedural fashions. Several works rely primarily on textual and pictorial content analysis of user profiles (e.g., Birnholtz et al., 2014; Chan, 2016; Miller, 2015) and thus can only draw conclusions regarding the content of self-presentations. Without narrative description through in-depth interviews, any conclusions drawn regarding motivations or techniques of impression management, as well as their antecedent factors, can only be conjectural. Adopting an interview-based based approach, we thus ask: *how do various elements of the social-technological ecosystem arising from GBMSM’s use of LBRTDAs, including affordances, communal common ground and the agent-structure dialectic, interact to shape users’ self-presentation behaviours?*

## Methodology

This research is part of a larger project in which 40 LBRTDA users across varying gender and sexual identities were interviewed about their use. Said project employed narrative inquiry to examine how LBRTDAs are transforming identities and social practices. We chose narrative inquiry because of its inherent potential to position participants’ subjectivities, illuminate examples of agency and cultural contestation, reveal human transformation and promote advocacy. Narrative inquiry also empowers participants by emphasizing their shared humanity through personal stories of joy, sorrows, struggles and the activities of daily living (Costa, 2005). Although the goal of narrative inquiry is to foreground the voices of participants, it is time-intensive and does not lend itself to large sample sizes or generalizability at the population level. Instead, it serves as an effective strategy for highlighting the complexity of participants’ experiences.

Participants were purposively selected to achieve diversity across gender (e.g. man, woman, trans, nonbinary) and sexual identity (e.g. lesbian, gay, bisexual, queer, straight) (see Table 1). Consistent with other works (Blackwell et al., 2015; Bonner-Thompson, 2017; Fitzpatrick and Birnholtz, 2018; Miles, 2017, 2019; Møller and Petersen, 2018), participants were recruited directly through LBRTDAs. Members of the research team created accounts on various apps and stipulated in their profile descriptions they were seeking to recruit participants. Similarly to Bonner-Thompson (2017), we opted to have team members display themselves in their profile picture rather than use a generic image, University logo or leaving the field blank, our reasoning being that this would encourage other users, especially those of diverse sexual and gender identities, to recognize us as ‘insiders’ rather than detached institutional operatives, thus incentivizing participation (Cuomo and Massaro, 2014). Being cognizant of these apps’ normative modes of use and the attendant potential for our intentions to be misconstrued (Birnholtz, 2018); a deliberate effort was made to present the researchers in ways that would disambiguate their ‘off-label use’ (Duguay, 2020), such as using pictures wherein they were fully clothed and assuming a more ‘professional’ pose and framing.

**Table 1.** Participant characteristics. All dimensions are taken verbatim from participants’ self-description.

Pseudonym	Age	Sex/gender	Sexual identity	Race/ethnicity	LBRTDAs used
Sajan	28	Male	Gay	Bangladeshi	Grindr
Linus	30	Man	Gay	White	Grindr, SCRUFF
Haroon	27	Man	Gay	South asian	Grindr, Tinder
Nicolas	24	Male	Attracted to masculine-identifying persons	Latino	Grindr
Ethan	26	Male	Gay or queer	White	Grindr, SCRUFF
Mitch	Generation X	Male	Gay	White	Grindr
Jackson	22	Cis male	Homoflexible	White	Grindr
Robert	26	Cis male	Gay	Black	Grindr
Nathan	Older end of the millennial scale	Cisgender male	Gay	White	Grindr, Recon
Benny	Millennial	Trans man	Gay	White	Grindr

When conducting the interviews, interviewers were matched as best as possible to participants according to their gender and sexual identity as to promote feelings of mutual trust and safety, and contribute to conversations that require ‘insider’ or emic knowledge. To ensure the interviews would generate rich and consistent data, we constructed a semi-structured interview guide organized around three research questions: (1) How are LBRTDAs influencing gender and sexual identities? (2) What impact (positive or negative) are LBRTDAs having on sexual relationships and overall quality of life? and (3) How do LBRTDAs shape and reconfigure public space? Interviews took place in person in a public, mutually agreed upon location and lasted between 1 and 3.5 h (mean 90 min). Participants received a $25 gift card in appreciation of their time. Interview quality was monitored through periodic checking of recordings and debriefing with interviewers.

For the purposes of this paper, we analyse the 10 interviews conducted with men who are gay, bisexual, queer or otherwise interested in other men. All included participants were current or previous users of Grindr and/or SCRUFF, though one participant primarily used Recon. Participants originate from the Greater Toronto Area, Canada, and as such mostly represent a mix of urban and suburban living.

Data were analysed in an inductive manner resembling constructivist grounded theory (Charmaz, 2014). Using NVivo, open coding was first performed to label relatively granular units of data – for example, on a line-by-line basis – with constructs mostly derived from participants’ own words. This approach was supplemented with axial and theoretical coding, whereby more significant and/or recurring codes were collated and juxtaposed against extant theoretical concepts to frame, extend and refine the central themes that form the core of our analysis. To ensure ‘groundedness’ in participants’ views and experiences, we undertook frequent memo-writing and comparison between data, emerging codes and theoretical concepts, only applying theoretical codes that properly ‘earned’ their way into the analysis (Thornberg, 2012). Trustworthiness (Shenton, 2004) was pursued by having two separate members of the research team independently read and code the transcripts and compare their codes. Although a multitude of themes were uncovered, we focus herein on those related to self-presentation.

## Findings and discussion

Participants identified a number of techniques and contingencies of self-presentation on GBMSM-targeted LBRTDAs, the overwhelming majority of which relate to the act of profile curation. As others have noted (Ellison et al., 2006), it makes sense to allocate a considerable portion of self-presentation efforts to the profile because it serves as the first point of contact and heavily influences whether and how one chooses to pursue further interaction. In our findings, we identify some of the major trends in impression construction as they relate to profile curation. We then consider factors influencing impression construction, including affordances, communal common ground and disposing elements of the structure-agent dialectic.

### Impression construction trends

Our analysis of determinants of impression construction on gay male LBRTDAs is served by a preliminary exploration of the modes of expression that predominate. Although we observed enormous variation in how participants deployed various expressive techniques, including choice in display picture framing, pose, dress, profile description subject matter, and fixed-choice identifiers (e.g., weight, height, body type, ‘looking for’, ‘tribe’), to name just a few examples, participants overall vacillated between two general self-presentation styles vis-à-vis the self-concept: authentic and self-enhancing display. Those endeavouring to present themselves ‘authentically’ did so by indexing their self-concept as accurately as they felt possible and with minimal contrivance. Sajan notes, for instance:

I feel like I should be truthful. Body type I say average. Position I say bottom because I am. I say that I’m single and I’m looking for dates, friends, networking, right now, all those things. I’m open about my HIV status, at least when I was last tested.

Present in similar measure was the desire to craft a specific image, based on audiences’ perceived values and expectations, that would accentuate one’s attractiveness, desirability or worthiness, that is, engage in self-enhancement. Self-enhancing displays were understood by participants as being constructed to some extent irrespectively of the self-concept, as illustrated by their frequent juxtaposing against authentic displays. Jackson explains:

I don’t like to lie. That said, I don’t like being 130 pounds either, but am I going to lie about being underweight? I don’t know. I never do. But that’s one thing I don’t like putting up there.

Jackson, interestingly, notes feeling compelled to present himself in a way that betrays his self-knowledge as to appear within the latitude of cultural acceptance regarding body weight, despite the moral value he places on truthfulness. Thus, unlike authentic displays, self-enhancing displays pose the possibility of deception when the audience values which one aims to exemplify clash with the traits one knows themselves to possess. The above excerpt also underscores that authentic and self-enhancing displays not only co-exist on the medium, but given their differences in the degree to which they accommodate or impel deception (here defined, irrespective of motive, as to some extent diverging from the self-concept), can present as duelling motives and therefore a point of tension within any individual user.

However, participants also located acts of deception within a hierarchy of severity, ranging from minor fudging of details like height and age to complete fabrication of personal identity (i.e., ‘catfishing’), which suggests authenticity and enhancement *qua* deception should be seen not as binary, mutually exclusive self-presentation choices but poles on a continuum. It is possible, as in Jackson’s case, to craft an impression that is mostly faithful to the self-concept save for some small embellishments – it would be reductive simply to label his real or fake.

Our findings echo previous research on heterosexual men and women’s use of social media and dating technologies that demonstrate, on balance, a propensity for authentic self-presentation which is punctuated by modest falsehoods and less frequently by blatant, totalizing deception (DeVito et al., 2018a; Ellison et al., 2006, 2011; Hancock and Toma, 2009; Ranzini and Lutz, 2017; Ward, 2017). However, any observed consistencies in the *relative prominence* of self-presentation styles across technologies and contexts of use could in theory belie differences in the assortment and patterns of interaction between factors involved in their construction. It is to these we now turn.

### Affordances’ influence on impression construction

Several elements of GBMSM-targeted LBRTDAs’ technological infrastructure were identified as making certain forms of self-presentation behaviour possible, with some facilitating and others constraining deceptive, self-enhancing display. Among the most influential in this regard, according to participants, is the relative lack of presentation flexibility (DeVito et al., 2017) when compared to face-to-face interaction. Many of the paralinguistic and nonverbal cues contained in one’s physical communicative repertoire (e.g., body language, facial expressions, vocal tone and pitch) are obscured in the primarily pictorial and text-based medium. As such, users are limited in their ability to develop a holistic impression of others prior to meeting in person. This is demonstrated in participants’ recurrent accounts of being taken aback in the transition to physical encounter by certain cues that went undetected in online interactions. Mitch recalled once meeting an individual whose manifest skittishness as reflected through their body language came as a complete surprise due to how well it was concealed online:

So I met this one guy ... I was driving and I had the app open ... and so he messaged me and we started chatting and that was an interesting conversation ... and I took a side trip and I actually met him in person ... It was very weird. He was not at all comfortable, which is too bad because he was really a nice guy ... He just, like, tensed up whenever I got close to him.

Gay male LBRTDA users elsewhere offer similar accounts of being jarred by the disparity between dates’ ‘real life’ presentation and online persona but nevertheless feeling obligated to complete a sexual contract that was implicitly agreed to in online communication (Miles, 2017).

LBRTDAs, like other technologies, thus seem to exemplify Walther’s (1996) notion that cue impoverishment in electronic communication enables more strategic self-presentation by reducing the range of expressions performers must monitor, control and refine. Incidentally, and much to the performer’s benefit, the majority of these missing expressions are those that would typically be ‘given off’ in face-to-face interaction and otherwise discredit a performance. Hence, should one opt to make self-enhancing displays via deception there is reduced threat of their being undermined in due course by one’s own efforts. Robert affirms as much in positing text-based communication is a boon in particular to individuals who struggle during face-to-face interaction to successfully wield the entirety of their expressive front in service of making favourable impressions:

I think a lot of people who do use these apps are socially a little inept ... it’s a lot easier to ... communicate through written language. And texting is a sort of a written language.

The present findings suggest, if anything, that cues are even further restricted in variety and quantity on most GBMSM-targeted LBRTDAs compared to social media and traditional web-based dating sites, which lends to even more streamlined and optimized self-presentation. Most apps, like Grindr, allow for only a single display picture or very limited series of images, whereas on Facebook and other platforms users can curate whole albums containing potentially thousands of photos. Textual description is also usually restricted to a short personal bio and series of pre-set identificatory categories compared to matchmaking sites like OkCupid, which allow for construction of elaborate profiles with lengthy bios and responses to personal questions. Because most apps also lack any kind of visible list of network ties (e.g., friends, followers), personal media stream (e.g., wall or timeline) or public feedback system (e.g., comments and likes), there is little opportunity for *other* users to supply cues to one’s front, or engage in *co-construction* of impressions (Ellison and boyd, 2013). Given the salience of cues is inversely in proportion to their abundance, it is noteworthy the degree of inference often made in regard to others’ personality and circumstance based on minor elements of their profile. Nicolas offers a sense, for instance, of how personas can be constructed *de novo* from as little as one picture:

I think the most ideal [display picture] would be ... face in photo, with shirt off, in a somewhat tropical or mountainous location ... something exotic ... tan skin, dark hair ... well, you know the stereotypical, like, tall dark man or whatever people look for … maybe holding a dog. Doing something that makes him look a little bit more down-to-earth, so, as though they’re, like, laughing in a candid photo.

Nicolas demonstrates how the imagination is apt to wander in digital contexts where counterfactual cues are readily suppressed. Those with a solid grasp of the cues that remain and the imagined realities they index are therefore presented with a unique signifying economy ripe for capitalization.

### Common ground that influence impression construction

A central idea of the affordance perspective is that altering the material features of a technology alone is not enough to guarantee that users will carry out a certain intended line of action. Even though cue reduction makes dissimulation possible in theory, users are only inclined to misrepresent themselves insofar as such behaviour is sanctioned by a given digital space’s communal common ground. One such norm which generally works in tandem with cue reduction to facilitate deception is the diffuse expectation or belief that the majority of other users are engaging in some degree of self-enhancement contra to their self-concept. With time and experience (e.g., through extended conversation, sharing of additional photos or meeting in person) users seem to develop an appreciation of the potential afforded by the technology for strategic self-presentation and others’ penchant for taking advantage of it. A seasoned user who has met enough individuals off Grindr to get a basic sense of any patterned disparities between online and offline selves, Mitch concludes,

The way I look at it is that nobody is as good looking as they say they are [on LBRTDAs] ... nobody’s as successful as they think they are ... But I think people have this expectation when they go on an app that they’re going to be able to make a connection with somebody, when the reality of it is that it’s all a little bit smoke and mirrors.

Although similar sentiments have been documented at the height of traditional web-based dating sites’ popularity (Ellison et al., 2011; Fiore and Donath, 2004), participant narratives indicate that increases in the sophistication and accessibility of self-enhancing technologies like photo retouching software in the intervening years (see Chua and Chang, 2016; Hess, 2015) have contributed to these assumptions being more salient on LBRTDAs.

As Mitch’s somewhat disparaging tone would suggest, however, belief in the normalcy of lying on LBRTDAs does not equate to personal tolerance or approval per se. Consistent with prior work (Toma and Hancock, 2010); our participants were generally disapproving of lying as a matter of principle. To understand why perception of preponderance should encourage deception despite widespread moral opposition, we must consider how this norm works in synergy with others – perhaps most plainly, the marketplace ideology endemic to most LBRTDAs which encourages self-objectification and competitiveness among users. Participants repeatedly likened the experience of navigating Grindr’s interface to perusing an e-commerce site or brick-and-mortar retailer – large quantities of similar ‘products’ are neatly displayed so that one might make thorough and measured determinations of cost-benefit before deciding between alternatives. This context was suggested to instill in users a sense of imperative to differentiate themselves from their contemporaries in order to capture consumers’ limited attention. To that end, self-presentational embellishments can offer a competitive advantage against those who present themselves in a more unvarnished manner. In support of this point, Sajan offers a detailed description of the self-presentation techniques that make him a ‘good marketer’, which tellingly include accentuating and even misrepresenting certain features:

The way Grindr is set up is that you don’t see profiles. You see a grid of faces. It’s very visual. And just the way people’s brains are set up, they would kind of go towards what they like the most. You’re going to be blown away by how much of a good marketer I am. So, I recently updated my profile picture, coiffed my hair to make it perfectly straight into a pompadour ... half of my face was lit by the sunlight that was coming in, and made my eyes kind of, like, look brown instead of black.

Hence, expectation of deception may encourage such behaviour not necessarily by fostering personal acceptance, but by introducing a degree of pressure to conform and compete within a socio-sexual economy of oversupply. Other researchers have similarly posited that LBRTDA dynamics exhibit many of the hallmarks of mercantilism, including rational exchange, reductionism, self-optimization and personal enterprise (Goldberg, 2020; Licoppe et al., 2016; Roach, 2013). Within this system of relations, self-enhancement can readily be justified as responding to market pressures.

Other elements of common ground on GBMSM-targeted LBRTDAs work to constrain or disincentivize deceptive, self-enhancing displays. Among the most pronounced is the expectation of eventual transitioning from online conversation to offline, face-to-face interaction. Indeed, so normatively accepted is this mode of practice that deviation tends to elicit suspicion of untoward motives. Nicolas even suggests a de facto time limit in which users are expected to meet before the possibility for relationship development is foreclosed upon:

That can take an hour, that can take a week [to transition from interacting on LBRTDAs to in person] ... but ... if you do not meet within the first couple weeks, I’d say 2 weeks max, of having conversation, you won’t meet. The conversation is going to die off because it’s just an online conversation and it didn’t lead anywhere.

Nicolas’ point underscores, moreover, that LBRTDAs are normatively perceived as means to a very specific end – that being physical encounter. Conversation for its own sake without any serious prospect of in-person connection is seldom appreciated or actively pursued. Further demonstrating the extent to which this particular mode of use has purchase over alternatives, Miles (2019) found those who seem to forestall the online-offline transition are maligned as ‘time-wasters’ who ‘misunderstand’ the purpose of such apps.

The omnipresent pressure of physical encounter constrains misrepresentation because it sensitizes users to the possibility that any embellishments, omissions or lies in their online presentation will be found out upon re-entry to the full-cue environment. For most, the imminent threat of sanctioning in face-to-face interaction acts as a strong deterrent against any kind of deception beyond what is normatively acceptable. This logic is evidenced in the curatorial decisions of Benny, who felt a sense of obligation to be forthcoming in the early stages of conversation that his genital morphology deviates from cisnormative standards of sex/gender and embodiment:

[There are] so many different ways that I have put on my profile to portray that I am trans to people. I switch between ‘FTM’ or ‘trans man’, or like, even just ‘man with a pussy’. But sometimes, that doesn’t even lead them down the path to understanding that I am a man … with a pussy ... but there is a big class divide on Grindr where it’s, like, if you’re transgender ... if you’re, you know, someone who used to be female, then you’re not quite a man, and you’re expected to advertise that.

The above provides a useful illustration of how multiple, opposing factors can interact to shape impression construction. LBRTDAs afford Benny the means to strategically construct his self-presentation in a way that elicits favourable reactions from audiences by concealing the ways in which his embodied self deviates from normative expectations. However, Benny uses LBRTDAs with the intention of eventually meeting his conversational partners, some of whom he expects would censure him (or worse) were he not adequately truthful. In a manner consistent with several other trans participants in a recent study by Fernandez and Birnholtz (2019), concerns of safety override the desire for optimized digital self-presentation and encourage ‘proactive display’ of his trans status.

While other researchers have similarly argued that anticipation of future in-person interaction curtails the impulse to lie in online dating (Birnholtz et al., 2014; Ellison et al., 2006; Guadagno et al., 2012; Hancock et al., 2007; Ward, 2017), our findings indicate this expectation more inexorably structures interaction on LBRTDAs than previous technologies. One could go as far as to say that brokering rapid physical connection is LBRTDAs’ *raison d’être* (Miles, 2018)*,* whereas in traditional web-based services greater emphasis was placed on sociality *within* the technology. Thus, on LBRTDAs the prospect of physical encounter could contribute to even further skew towards authentic display. That said, apps’ inherent logics do not dictate modes of use, and presentation authenticity might vary by user motive or kind(s) of relationship sought. For example, someone seeking a long-term relationship might have greater incentive to be truthful than someone seeking a casual hookup because dissimulation carries higher personal costs in the former case.

### Influence of agent and structure on impression construction: The case of stigma

As mentioned above, users’ social location is likely to influence how they perceive and wield a medium’s affordances to achieve their self-presentational goals. GBMSM are unified in their possessing a stigma, that is, a trait which a priori deviates from normative expectations of how individuals *should* be and therefore discredits possessors’ self-image upon revelation to certain audiences. Hence, for GBMSM successful self-presentation is at times reliant on concealment of the trait in question – in this case, nonheterosexual desire, activity and identification. On platforms with more generalized userbases and modes of use, such as Facebook or Twitter, this can be achieved relatively straightforwardly by compartmentalizing indicators of sexual identity while presenting oneself authentically in most other respects. On dating technologies, however, and particularly those less ‘porous’ to use by heterosexuals (Ferris and Duguay, 2020), membership in and of itself can be an indicator of sexual identity. As our findings suggest, this often requires stigma management be performed by way of reducing one’s identifiability outright – either by obscuring personalizing attributes (e.g., name, face, location) or fabricating whole personas.

By virtue of their stigmatization, we would expect to see, overall and other things being equal, higher rates of deception by GBMSM on dating technologies compared to heterosexual men. An incipient literature suggests this might indeed be the case – Ranzini and Lutz (2017) found, for instance, that GBMSM were more likely to engage in deception than their heterosexual counterparts on Tinder. It would be inaccurate, however, to suggest that mere possession of a stigma uniformly predisposes this group to deception. After all, participants in our own study, all self-identifying GBMSM, exhibited considerable variation in presentation authenticity. It seems, rather, that individuals’ life circumstances, including the composition and organization of their social network ties in terms of awareness and acceptance of their sexual identity, determine the degree of pressure one feels to strategically manage indicators of stigma through their online self-presentation. Stated differently, the way in which cis-heterosexism pervades the social structures that locate the subject present different pragmatic consequences for expression of nonheterosexual identity, hence incentive for concealment. Sajan, for example, observes a trend whereby individuals who are not ‘out of the closet’ (i.e., have not disclosed their nonheterosexual status to a majority of members of their networks or to key audiences (e.g., family members, employers)) are more likely to engage in deception via obfuscating their identity:

In a weird way, those who are not out of the closet or those who are, like, maybe in a relationship ... tend to be more vague... They probably have very little information on the profile, right? So that’s an example in which the closet would affect it — how they state their preferences and reveal information about themselves. ... A lot of them don’t have profile pics …[or] profile descriptions at all.

Concerns of stigma management serve not only to illustrate how various facets of technology use act *additively* to shape impression construction, however – their joint effects are also *multiplicative* in that the influence of any one element on self-presentation behaviour can be dependent on others. As a case in point, consider the affordances of audience transparency (‘the extent to which a platform affords user awareness of who is actually in the audience for persona-linked content’ (DeVito et al., 2017: 744)) and visibility control (‘the extent to which a platform affords individual determination of what content linked to their persona is visible to others’ (DeVito et al., 2017: 744)), which together define users’ ability to know and exert control over who can see their expressions online. Generally speaking, participant responses suggest audience transparency and visibility control are low on gay male LBRTDAs compared to most social media platforms. Unlike sites like Facebook or Twitter, where users can employ a number of privacy controls to limit the size of their audience, on LBRTDAs profiles are by default accessible in their entirety by anyone within a certain geographic range who creates an account. Users are therefore restricted in the way of audience control to blocking others on an individual basis – in other words, by strategies of ‘opt out’ rather than ‘opt in’ (Blackwell et al., 2015). In itself, however, audience transparency/control does not appear to consistently drive users one way or the other vis-à-vis presentation authenticity. A key determining factor is whether there is an appreciable risk of one’s expression of nonheterosexuality, as implied by their presence on GBMSM-targeted LBRTDAs, reaching audiences of personal significance who would derive a negative impression. Though the specter of unknown audiences becoming privy to one’s same-sex leanings was raised as a possible motivator for deception, this did little to deter participants who were ‘out’ to the majority of their contacts from identifying themselves openly and honestly. Linus explains:

I’ve never been worried about being recognized, because I’ve always put my face picture on…I was out and I wasn’t worried about people finding out…I see people I know all the time on there, especially classmates or people who are a year above me at school, people who are potentially professional connections. So I don’t worry [as] I have links to my different social media. So yeah, my personal details are pretty much open.

In other words, proper audience visibility/control is prerequisite to authentic display of sexual identity more so among those for whom nonheterosexuality still constitutes a form of ‘destructive information’ (Goffman, 1959) that would discredit them in the eyes of their most routine and/or significant audiences. In support of this notion, prior research demonstrates LGBTQ+ social media users who remain connected to significant others that presumably would stigmatize their sexual identity employ various visibility control measures to re-segregate audiences, including tailoring privacy features and friend lists (Duguay, 2016) and distributing content strategically across platforms (DeVito et al., 2018b).

One additional affordance whose influence on impression construction seems to depend on experiences and concerns relating to stigma management is locatability. An affordance that is characteristically prominent on LBRTDAs, locatability refers to the potential for users to acquire information about others’ geographic location or to transmit their own (Schrock, 2015). Those who anticipate minimal social repercussion from others being made aware of their nonheterosexual identity viewed LBRTDAs’ hybridizing of physical and digital space (Miles, 2018) mostly in terms of possibility for fostering connection. Jackson, for instance, extols Grindr’s precise distance markers for aiding in identifying other GBMSM in physical venues:

Grindr is a great gaydar. That’s another reason I do have it ... so basically when I opened up Grindr at [the local gay bar] when I was partying, suddenly I knew everyone’s name, suddenly I knew, like, faces. I’m like, oh, you’re this person, you’re that person.

Conversely, for those who see their identifiability on GBMSM-targeted LBRTDAs as posing a threat to their fostered self-image, location data represent another form of personalizing information that warrant concealment or strategic disclosure. Mitch explains

I’ve always been a little uncomfortable with the proximity-distance thing. ... It’s a privacy thing. I mean, the one thing I do like about the apps is that they provide a level of privacy and anonymity to it, and adding in that distance function, to me, violates that in my mind.

Miles (2019) similarly observed that individuals who are not yet ‘out’ are more hesitant to embrace the locative/hybridizing function of LBRTDAs. The present findings expand on this by demonstrating that hybridity also contributes to deception among this contingent of users in particular.

Finally, persons’ positioning within general social structures can also modulate the ways in which the communal common ground of LBRTDAs impel certain presentation strategies. Nicolas’ above description of the ‘ideal’ profile picture which evokes a particular racialized and embodied subject (‘something exotic ... tan skin, dark hair’) speaks to the persistence of norms among this demographic of users that allocate value across categories of sociopolitical difference. GBMSM’s common possessing of a sexuality-based stigma does not preclude their reinforcing in the collective imaginary a series of social hierarchies based on a number of additional intersecting stigmas, including but not limited to those towards Blackness, nonwhiteness, effeminacy, transness, disability, serostatus and fatness. Critically, where one figures within these hierarchies – the stigmas they accrue which undermine their ability to generate a positive impression and reap the attendant relational spoils – shapes the pressure they feel to deceive. Jackson conjectures

I find people who are a person of colour on Grindr tend to express less on their profile, they’re less likely put up a photo ... they don’t chat as much. That’s probably because of the stigma and fear ... you don’t want to be harassed or anything like that.

Haroon affirms the preponderance of racist discrimination among users through description of an informal ‘experiment’ he performed to determine whether his race was the primary factor leading to his repeatedly being ignored:

I conducted an experiment. What I did was I used a picture of this television actor from India, and a good-looking one, but he didn’t get responses. Poor guy. More responses than I do get, but maybe one or two more ... they’re just not into brown skin.

Thus, those whose self-image deviates prohibitively from the normative ideal instantiated by gendered, racial and other power structures understandably see greater strategic merit in dissimulation.

## Conclusion

To reiterate, the purpose of the current study was to explore how various elements of GBMSM’s use of LBRTDAs, including affordances, communal common ground and the agent-structure dialectic, interact to influence self-presentation behaviour. In line with previous research (DeVito et al., 2018a; Ellison et al., 2006, 2011; Hancock and Toma, 2009; Ranzini and Lutz, 2017; Ward, 2017), we found impression construction on GBMSM-targeted LBRTDAs reflects tensions between authentic depiction of the self-concept and self-enhancement *qua* deception. In part, individuals’ position on this continuum was a result of the additive effect of various determinants, some facilitating of deception, including reduced cues, belief in the normalcy of lying, norms of self-objectification and competitiveness and concerns of managing sexual identity-related stigma, and others constraining, including expectation of brokering physical connection. Impression construction determinants also to some extent interact multiplicatively, that is, in a way where the influence of one is dependent on another. This was most plainly evidenced in the interactions between stigma management concerns, the affordances of audience visibility/control and locatability and common ground reinforcing social hierarchy. Although several of these factors were previously identified within traditional web-based dating sites and heterosexual LBRTDAs, our findings suggest possible changes to their salience in this particular context of use. Importantly, the present study is by no means meant to be an exhaustive survey of the technological, structural and individual factors involved in impression construction; rather, it is intended to serve as an illustration, using impression authenticity as an exemplar, of the complex and recursive ways in which these factors interact in mediated display of identity.

Some limitations inherent to our choice in methods should be acknowledged. As previously mentioned, by using self-report data, we were able to gain insight into individuals’ motives and reasoning behind self-presentation decisions. However, this type of data is vulnerable to social desirability effects, particularly as they concern misrepresentation – as others have noted, participants are likely apprehensive to be totally forthcoming about how often and to what extent they lie (Ellison et al., 2006; Toma et al., 2008). Additionally, by restricting our focus to LBRTDA use we limit our ability to understand how these technologies figure into users’ self-presentational choices across their broader social media ecosystem (DeVito et al., 2018b). It is possible that individuals treat Grindr as a ‘back stage’ where they can present their nonnormative sexual identity with abandon whilst maintaining a purely heterosexual front for audiences on Facebook or LinkedIn. Furthermore, the lack of evidence of any departure from the self-concept motivated by a desire for identity experimentation or other forms of creative play could be an artefact of the present symbolic interactionist frame and methods deriving therefrom, including the line of questioning and analytic foci.

Finally, it should be recognized that the self-presentational strategies and contingencies detailed herein are based on description from individuals whose app use mostly conforms to that sanctioned or prescribed by the technology itself, that is, connecting for physical intimate encounter. This, however, falls short of capturing the gamut of user practises, eliding, among other possibilities, engaging in sex work, coordinating social gatherings, selling drugs, marketing, promoting social campaigns and having phone/cybersex (not to mention seeking research participants) (Duguay, 2020). Theoretically, these motives for use could impel different self-presentation strategies (e.g., intimation or supplication over enhancement) or affect the salience of various elements that locate the authenticity/deceptiveness of displays (e.g., expectation of offline encounter). The self-presentational implications of these various forms of ‘off-label’ app use hence merit future study.

Limitations notwithstanding, the present study marks the first attempt at systematically exploring how determinants of impression construction interact in the context of GBMSM-targeted LBRTDAs. This work is foremostly distinguished by its application of the symbolic interactionist frame to demonstrate how a diversity of self-presentational phenomena emerge from the complex and socially situated interactions between users’ goals, values, predilections, capacities and circumstances. It is also distinguished through its use of interviewing to render these underlying contingencies explicit. Ours joins a growing body of work that attest to the importance of giving due consideration to the socially-constructed character of communication technologies, demonstrating how the lived experiences of GBMSM complicate conventional understandings of self-presentation behaviour in digital space. As dating technologies further develop and integrate themselves into everyday practices of social and intimate connection, a more focused eye for such contextual variation will be required to accurately and thoroughly account for the changes taking place.
